# Visualizing the Bayesian 2-test case: The effect of tree diagrams on medical decision making

**DOI:** 10.1371/journal.pone.0195029

**Published:** 2018-03-27

**Authors:** Karin Binder, Stefan Krauss, Georg Bruckmaier, Jörg Marienhagen

**Affiliations:** 1 Mathematics Education, Faculty of Mathematics, University of Regensburg, Regensburg, Germany; 2 Institute for Primary Education, School of Education, FHNW University of Applied Sciences and Arts Northwestern Switzerland, Liestal, Switzerland; 3 University Hospital Regensburg, Regensburg, Germany; University of South Alabama Mitchell Cancer Institute, UNITED STATES

## Abstract

In medicine, diagnoses based on medical test results are probabilistic by nature. Unfortunately, cognitive illusions regarding the statistical meaning of test results are well documented among patients, medical students, and even physicians. There are two effective strategies that can foster insight into what is known as Bayesian reasoning situations: (1) translating the statistical information on the prevalence of a disease and the sensitivity and the false-alarm rate of a specific test for that disease from probabilities into natural frequencies, and (2) illustrating the statistical information with tree diagrams, for instance, or with other pictorial representation. So far, such strategies have only been empirically tested in combination for “1-test cases”, where one binary hypothesis (“disease” vs. “no disease”) has to be diagnosed based on one binary test result (“positive” vs. “negative”). However, in reality, often more than one medical test is conducted to derive a diagnosis. In two studies, we examined a total of 388 medical students from the University of Regensburg (Germany) with medical “2-test scenarios”. Each student had to work on two problems: diagnosing breast cancer with mammography and sonography test results, and diagnosing HIV infection with the ELISA and Western Blot tests. In Study 1 (N = 190 participants), we systematically varied the presentation of statistical information (“only textual information” vs. “only tree diagram” vs. “text and tree diagram in combination”), whereas in Study 2 (N = 198 participants), we varied the kinds of tree diagrams (“complete tree” vs. “highlighted tree” vs. “pruned tree”). All versions were implemented in probability format (including probability trees) and in natural frequency format (including frequency trees). We found that natural frequency trees, especially when the question-related branches were highlighted, improved performance, but that none of the corresponding probabilistic visualizations did.

## Introduction

Physicians, medical staff, and patients frequently have difficulty understanding what medical test results really mean. This is a serious issue because patients must often make tough decisions about specific medical treatments, for example after a positive test result from a routine screening [1]. Unfortunately, not only patients but also physicians and medical staff are often unable to combine and understand statistical information correctly. The resulting cognitive illusions can lead to an overestimation of the benefits of diagnostic methods or to an underestimation of the possible damage they could do [2,3]. For example, a positive HIV test result can lead to mental disorders or even suicide [4,5]. But what does an HIV test result really mean? Most counselors in the studies from Prinz et al. [6], Gigerenzer et al. [7], and Ellis and Brase [8] operate under an illusory belief that positive test results indicate certainty. But in fact, a positive HIV test result does not indicate the presence of HIV infection with absolute certainty [9].

Of course, the same applies to other medical diagnostic procedures. Another example is the mammography screening for breast cancer, which is very expensive and heavily promoted in many countries as necessary for every woman in a particular age group [10]. In the following, we call judgments based on a single medical test *1-test cases*.

## The medical 1-test case

A study by Eddy [11] shows that even physicians are often unable to combine the statistical information of a breast cancer screening diagnosis in a 1-test case correctly. For instance, consider a situation in which breast cancer is diagnosed based on a mammogram (adapted from [11]):

Screening for breast cancer—1-test case (Probability Format):

The probability of breast cancer is 1% for a woman of a particular age group who participates in a routine screening. If a woman who participates in a routine screening has breast cancer, the probability is 80% that she will have a positive mammogram. If a woman who participates in a routine screening does not have breast cancer, the probability is 9.6% that she will have a false-positive mammogram.

What is the probability that a woman who participates in a routine screening and has a positive mammogram has breast cancer?

In the situation above, the *a priori probability* P(B) = 1% denotes the prevalence of the disease in a particular age group. The conditional probabilities P(M+|B) = 80% and P(M+|←B) = 9.6% are called the *sensitivity* and the *false-alarm rate* of the mammography. In medicine, the *a posteriori probability* P(B|M+), which is the relevant one for patients, is called the *positive predictive value* of a medical test. The Bayes’ theorem shows that the actual probability of breast cancer given a positive mammogram P(B|M+) is only about 7.8%.

$$\begin{array}{c} P(B|M+)=\frac{P(M+|B)∙P(B)}{P(M+|B)∙P(B)+P(M+|\neg B)∙P(\neg B)}\\ \;=\frac{80\%∙1\%}{80\%∙1\%+9.6\%∙99\%}\approx 7.8\% \end{array}$$

However, most physicians in Eddy`s study assumed this probability to be between 70% and 80%, far from the correct positive predictive value. A wide variety of empirical studies have shown that physicians, medical staff, and patients [12,13] have difficulties with problems of this kind. Furthermore, Bayesian reasoning problems are of relevance in many other domains, and the respective cognitive illusions are well documented among school students [14], university students [15], legal professionals [16], and managers [17].

Fortunately, there are two highly effective strategies for overcoming occurring cognitive illusions and helping people to understand statistical information—namely, natural frequencies and visualizations.

### Strategy 1: Natural frequencies instead of probabilities

Rather than presenting all statistical information in the format of confusing conditional probabilities and percentages, one can provide natural frequencies as a means of describing Bayesian reasoning situations. In a seminal paper, Gigerenzer and Hoffrage [18] translate the numbers in the breast cancer screening problem into natural frequencies:

Screening for breast cancer—1-test case (Natural Frequency Format):

100 out of 10,000 women of a particular age group who participate in a routine screening have breast cancer. 80 out of 100 women who participate in a routine screening and have breast cancer will have a positive mammogram. 950 out of 9,900 women who participate in a routine screening and have no breast cancer will have a false-positive mammogram.

How many of the women who participate in a routine screening and receive positive mammograms have breast cancer?

It is now easier to see that there are 80 + 950 women with positive mammograms, and that only 80 out of these 1,030 women actually have breast cancer, which again results in a positive predictive value of about 7.8%. With the natural frequency version significantly more people are able to make the correct inference [18,19], because one simply needs to calculate the proportion of women with breast cancer among those who have a positive mammogram.

For more than 20 years, natural frequencies have been a well-known tool for overcoming cognitive illusions in Bayesian reasoning situations, also with respect to slightly more complicated scenarios, such as the notorious Monty Hall problem [20]. More generally, frequency formulations (beyond natural frequencies) have also been able to reduce the so-called conjunction fallacy (see, e.g., the Linda Problem [21,22]). With regard to Bayesian reasoning, there are myriad studies showing the enlightening properties of natural frequencies in a variety of domains: they help physicians in diagnostic inferences [12,13], patients in understanding these diagnoses [13], advanced law students in adequately evaluating legal indications [16], and managers and executives in management decisions [17], as well as university students [23] and secondary school students [14]. Even fourth graders are able to solve Bayesian reasoning tasks using natural frequencies [24].

A recently conducted meta-analysis from McDowell and Jacobs [25] reviews the results of 35 papers describing the impact of natural frequencies on decision-making processes and finds that the facilitating effect of natural frequencies is quite robust; the estimated average percentage correct for the probability versions of Bayesian reasoning tasks is 4%, while it is 24% for the corresponding natural frequency versions. Although there has been some discussion concerning the beneficial effect of natural frequencies [26,27], this effect has generally been recognized [25] and repeatedly replicated by now (for an exception see [28]), because they simplify the Bayesian calculation and more people are able to find the correct solution.

### Strategy 2: Visualizing Bayesian reasoning tasks

**Fig 1:**
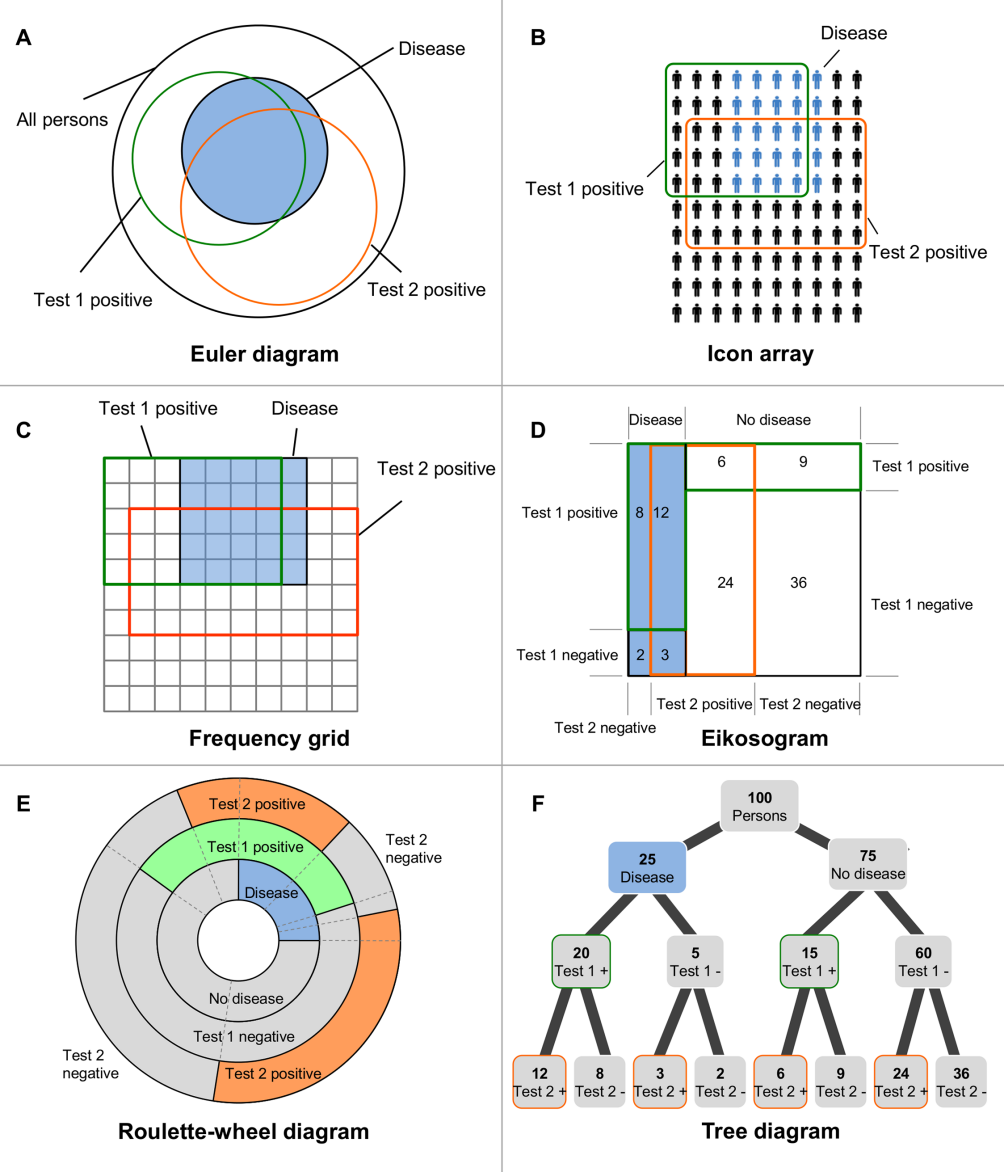
Six different types of visualization for the Bayesian 2-test case. (A) Euler diagram (B) Icon array (C) Frequency grid (D) Eikosogram (E) Roulette-wheel diagram, and (F) Tree diagram. Omitting the information on the second test in the different visualizations results in the corresponding visualization of the 1-test case.

There is another strategy for improving Bayesian reasoning in the 1-test case, namely, visualizing the statistical information. Some prominent visualizations that have been developed are *Euler diagrams* (e.g., [29–31]), *roulette-wheel diagrams* (e.g., [32,33]), *frequency grids* (e.g., [23,34,35]), *Eikosograms* (sometimes also called *unit squares* or *mosaic plots*; e.g., [36–39]), *icon arrays* (e.g., [32,40,41]), *2×2-tables* (e.g., [14,42]), and *tree diagrams* (e.g., [14,33,42–44]). For an overview of these visualizations, see [14], and for corresponding visualizations regarding the 2-test case, see Fig 1. With respect to the first strategy (natural frequencies), it must be noted that most visualizations do not contain any numbers (e.g., icon arrays, frequency grids, roulette-wheel diagrams or Euler diagrams) and therefore can illustrate natural frequency or probability versions as well.

Several of these visualizations have already been tested empirically (for an overview, see [14,45,46]). The previously mentioned meta-analysis [47] found that visualizations can also improve participant performance in Bayesian reasoning situations. The aggregate effect across various visualizations is an increase in correct inferences of about 23 percentage points. However, there is evidence that not all types of visualizations support people in their decision-making processes. With visualizations that contain numbers (i.e., tree diagrams or Eikosograms), the format of these numbers can make a difference in how participants understand the statistical information. For instance, it must be noted that in the 1-test case, only tree diagrams containing natural frequencies in the nodes, not tree diagrams with probabilities at the branches [14,23] or without any numerical information [43], significantly foster insight into Bayesian reasoning problems.

## The medical 2-test case

So far, empirical studies concerning visualizations of Bayesian reasoning situations are predominantly conducted with 1-test cases (for visualizing cases with non-binary hypotheses, see [32,33,48]). However, in many medical real-life applications, there is more than one medical test (or clinical symptom) available [49].

For instance, consider a situation in which breast cancer is diagnosed based on both a mammogram and a sonogram (adapted from [50,51]):

Screening for breast cancer—2-test case (Probability Format):

The probability of breast cancer for a woman of a particular age group is 1%. The probability that a woman with breast cancer will have a positive mammogram is 80%. The probability that a woman with breast cancer will have a positive sonogram is 95%. The probability that a woman without breast cancer will have a false-positive mammogram is 9.6%. The probability that a woman without breast cancer will have a false-positive sonogram is 7.8%.

What is the probability that a woman with a positive mammogram and a positive sonogram actually has breast cancer?

For alternative ways to present the statistical information of 2-test cases, for example by providing a *combined* sensitivity and a *combined* false-alarm rate, see the S1 Appendix. In the following we apply both natural frequencies *and* visualizations to situations where two medical test results are provided.

### Strategy 1: Natural frequencies

Just as in the 1-test case, diagnoses based on two indicators can be formulated with natural frequencies instead of probabilities. Translating the 2-test case described into a natural frequency format yields:

Screening for breast cancer—2-test case (Natural Frequency Format):

100 out of 10,000 women of a particular age group have breast cancer. 80 out of 100 women with breast cancer have a positive mammogram. 76 out of 80 women with breast cancer and a positive mammogram have a positive sonogram. 950 out of 9,900 women without breast cancer have a false-positive mammogram. 74 out of 950 women without breast cancer but with a positive mammogram have a false-positive sonogram.

How many of the women with a positive mammogram and a positive sonogram actually have breast cancer?

It has already been demonstrated empirically that the beneficial effect of natural frequencies is not limited to Bayesian 1-test cases but also holds for 2-test and even for 3-test cases [50,51]. Furthermore, Hoffrage et al. [51] successfully applied the natural frequency strategy to situations where either three hypotheses (e.g., disease A, disease B, or healthy) or three test results (e.g., positive, negative, or unclear test result) were provided. Yet as far as we know, only strategy 1, not strategy 2 (applying visualizations), has been investigated with regard to 2-test cases.

### Strategy 2: Visualization

Generally, all visualizations of “simple” Bayesian reasoning problems (i.e., one binary hypothesis must be inferred from one binary cue) can be extended to visualizing medical 2-test cases (see Fig 1). It is not immediately obvious, however, which visualization is most helpful in 2-test cases. In the following we will point out why we chose to study tree diagrams.

## Some general remarks on visualizing Bayesian reasoning problems

There are basically two possible applications of visualizations (regardless of the number of tests provided): (1) Visualizations can be *presented* to illustrate statistical information for physicians or patients. One can present visualizations *in addition to* textual information or *instead of* textual information. It is an open question as to which of these variants is most helpful for understanding the situation. (2) If no visualization is provided, problem solvers could *create visualizations on their own* in order to understand the situation. Here the question of which visualization can be produced with the least amount of effort arises.

Thus, it would be advantageous if the visualization were not only cognitively helpful but could also be constructed quickly simply using paper and pencil. Regarding Fig 1, producing Euler diagrams (Fig 1A), frequency grids (Fig 1C), Eikosograms (Fig 1D), and roulette-wheel diagrams (Fig 1E) all obviously require deliberate geometrical operations. Concerning Euler diagrams (Fig 1A) and roulette-wheel diagrams (Fig 1E), even areas of circles or circle sections have to be constructed. And with the icon array (Fig 1B), it is very tedious work to depict all of the figures (for N = 1,000 persons, 1,000 icons have to be charted). Furthermore, the geometrical nature of visualizations A-E (Fig 1) leads to the problem that extreme base rates (which are often responsible for cognitive illusions) are nearly impossible to depict. For example, in order to illustrate a base rate of 0.1%, visualizations such as A, C, D, and E (Fig 1) would contain unmanageably small areas, while icon arrays (Fig 1B) would require 1,000 symbols, thus all entailing enormous effort to produce these visualizations.

**Fig 2:**
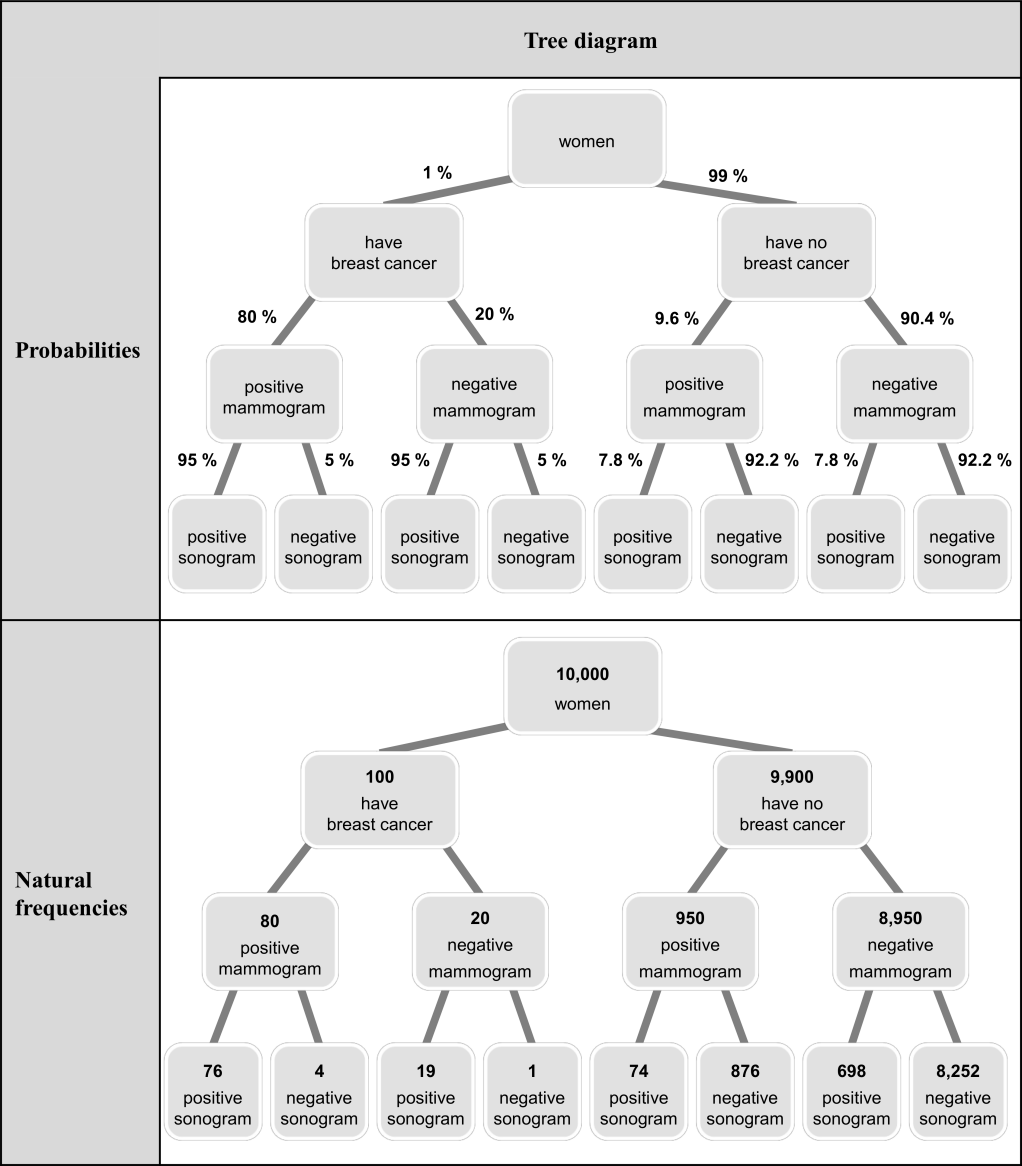
Probability and natural frequency tree of a 2-test case (implemented in studies 1 and 2).

In contrast, the tree diagram (Fig 1F) can be produced with a simple paper-and-pencil-operation in a short amount of time. Because the tree diagram is the only non-geometrical visualization, even very small base rates can be illustrated simply by depicting the respective numbers. In addition, tree diagrams generally can be equipped with both (conditional) probabilities at the branches (a strategy that is predominantly implemented in teaching statistics in secondary schools and at universities) and also natural frequencies in the nodes. Fig 2 shows tree diagrams with respect to both information formats, depicting a medical 2-test case (diagnosing breast cancer based on a mammogram and a sonogram).

Furthermore, there is another notable feature in tree diagrams that argues for choosing them for our empirical study: if the aim of the visualization is to illustrate the typical *conditional* probabilities of Bayesian reasoning tasks, the tree diagram is the only (!) possibility for visualizing the numbers of both the frequency format and the probability format. Let us explain this argument regarding the Eikosogram (Fig 1D), where the implemented numbers are frequencies (the sum of all numbers is 100). Of course these frequencies can be replaced by probabilities by simply adding the percent symbol after every number (if the sum, say N, were unequal to 100, probabilities could be derived by dividing by N). Yet it has to be noted that these eight percentage points then display *conjoint probabilities* but not *conditional probabilities*, which are predominantly displayed in Bayesian reasoning tasks (compare the versions above).

Interestingly, there is no intuitive way to display conditional probabilities in any of the other diagrams because there is no branch (or similar prominent place) for them (where should conditional probabilities be placed in Fig 1A–Fig 1E?). Since the diagnostic information of medical tests is usually presented in terms of sensitivities and false-alarm rates (or specificities; see [52]), this is a significant problem, especially if the problem solver has to construct the visualization on his or her own. This feature, namely that all numbers of typical Bayesian diagnostic situations can be directly implemented into tree diagrams, is an enormous advantage with respect to *teaching* statistics.

In addition, with reference to tree diagrams, it would be possible (and should be examined in a future empirical study) to provide probabilities in the branches (which are dominant in teaching statistics) and absolute numbers in the nodes simultaneously and therefore to present not only both formats, but also conjoint and conditional information in one visualization. With respect to Fig 2 this would mean adding the probabilities to the branches of the natural frequency tree or vice versa.

Note that all the arguments presented in favor of tree diagrams hold for 1-test cases as well as 2-test cases (or, of course, for cases with even more tests involved). In the following, let us focus on two details regarding the tree diagrams in Fig 2.

### Redundancy of information

It has to be noted that both the textual wording and the tree diagram already contain all of the information that is needed in order to solve Bayesian reasoning problems (given conditional independence; see S1 Appendix). Consequently, the question arises as to whether (a) only the wording, (b) only the tree diagram, or (c) both representations taken together best helps to solve the problem.

Cognitive Load Theory [53] and Cognitive Theory of Multimedia Learning [54] suggest that the representation of a textual wording in addition to a specific visualization might increase the extraneous cognitive load and thus might lead to poorer performance because of the redundancy principle [54]; however, the redundancy effect can reverse under certain conditions [55,56]. Similarly, results from a study of Micallef et al. [30] indicate that a visualization is only helpful when no (corresponding) textual information is additionally presented. In Study 1 we will address this issue of redundancy.

### Diagrams contain more information than the textual wording

A closer comparison of the statistical information presented in the tree diagrams (Fig 2) and the textual wordings reveals that the tree diagram contains *more* information than the textual wording. For example, while statistical information on persons with two negative test results are presented in the tree diagram, only statistical information on women with positive test results is provided in the text. Note that for the given question (“What is the probability of the disease given two positive test results?”), several branches of Fig 2 are indeed dispensable (for participant performance in alternative questions, see [57]). Thus it would be possible (a) to *highlight* both question-related branches or (b) even to *prune* the tree and only display those two relevant branches.

In cases (a) and (b), Cognitive Load Theory would suggest that according to the signaling principle, highlighting the relevant branches in the tree diagram (or even pruning the diagram by omitting the question-irrelevant branches) might improve participant performance [58,59]. However, the representation of unnecessary information could also increase the extraneous cognitive load; in that case, improved performance would be attained only with a pruned tree (since in a tree with highlighted branches the non-relevant branches would still be visible). Yet it has to be noted that only the full tree diagram allows the direct combining of numbers for *any* possible question that might be posed (e.g., “What is the probability of the disease given that test 1 is positive and test 2 is negative?” or vice versa). In Study 2 we focus on the issue of highlighting branches or pruning tree diagrams.

## Research question

It should be noted that with respect to all three following research questions, we will compare probability versions (including probability trees) with natural frequency versions (including frequency trees).

1. What is the effect of visualizing statistical information with a tree diagram in a Bayesian 2-test case (Study 1 and Study 2)?
2. Is it easier to solve a purely textual version, a purely visual version, or a version that presents the text and the tree diagram simultaneously (Study 1)?
3. Does it help to highlight relevant branches or even prune irrelevant branches instead of simply presenting a full tree diagram (Study 2)?

## Study 1

### Method

#### Participants

A total of 190 medical students (56 men, 133 women, one person who gave no answer) at different stages of their medical education at University Hospital Regensburg were recruited in 2016. Participants’ ages ranged from 18 to 41 years (*M* = 23.1, *SD* = 3.3). All students were informed that their participation was voluntary, and that anonymity was guaranteed. Participants had given their prior written consent to participating in the study. The Review Board of University Hospital Regensburg confirmed that, for this kind of study, no ethical approval would be necessary.

#### Design and materials

A paper-and-pencil questionnaire contained two successive Bayesian 2-test tasks. We implemented a 3×2×2 design with the factors *presentation of information* (text only vs. tree only vs. text and tree), *information format* (probabilities vs. natural frequencies) and *context* (breast cancer screening problem vs. HIV testing problem) (see also Table 1 and section “Procedure”).

**Table 1:** Design of the twelve resulting problem versions implemented (Study 1).

		Context	
		Breast cancer screening problem	HIV testing problem
**Information format**	Probabilities	**Presentation of information** • Text only • Tree only • Text and tree	**Presentation of information** • Text only • Tree only • Text and tree
	Natural frequencies	**Presentation of information** • Text only • Tree only • Text and tree	**Presentation of information** • Text only • Tree only • Text and tree

All versions began with a description of the medical situation (Table 2). After that, one of the six different presentations of information was provided. In the tree-only and text-and-tree versions, the tree diagrams of Fig 2 were implemented. Finally, the question was formulated in the same format as was used with the previous statistical information. The complete problem formulations can be seen in Table 2.

**Table 2:** Problem formulations for both contexts (breast cancer screening problem and HIV testing problem).

	Breast cancer screening problem		HIV testing problem	
	Probability version	Natural frequency version	Probability version	Natural frequency version
**Medical situation**	Imagine that you are a physician in a mammography screening center where women without symptoms are screened for breast cancer. In addition to mammograms, you frequently use sonograms as a supplementary medical test to detect breast cancer. At the moment, you are advising a woman who has no symptoms but who has received a positive result from her mammogram as well as a positive result from her sonogram. This woman wants to know what these results mean for her. For your answer, there is the following information available, which is based on a random sample of women who have all undergone a mammography and a sonography^1^:		Imagine that you are a physician in an AIDS information center. In addition to individual counseling interviews, your information center also provides HIV testing, for which two blood samples are taken: An ELISA test is conducted with the first blood sample. If the ELISA test is positive (indicating a possible HIV infection), a Western Blot test is ordered with the second blood sample. At the moment, you are advising a low-risk client who has received a positive result from the ELISA test as well as from the Western Blot test. This client wants to know what these results mean for him. For your answer, there is the following information available, which is based on a random sample of low-risk persons who have all undergone both the ELISA and the Western Blot test^1^:	
**Presen-tation of informa-tion**	• *Text only* • *Tree only* • *Text and tree*	• *Text only* • *Tree only* • *Text and tree*	• *Text only* • *Tree only* • *Text and tree*	• *Text only* • *Tree only* • *Text and tree*
**Text**	The probability of breast cancer for a woman with no symptoms is 1%. The probability that a woman with breast cancer will have a positive mammogram is 80%. The probability that a woman with breast cancer will have a positive sonogram is 95%. The probability that a woman without breast cancer will have a false-positive mammogram is 9.6%. The probability that a woman without breast cancer will have a false-positive sonogram is 7.8%. ^1^ Footnote: Assume for your calculations that the results of both tests are (statistically) independent for women with breast cancer as well as for women without breast cancer.	100 out of 10,000 women with no symptoms will have breast cancer. 80 out of 100 women with breast cancer will have a positive mammogram. 76 out of 80 women with breast cancer and a positive mammogram will have a positive sonogram. 950 out of 9,900 women without breast cancer will have a false-positive mammogram. 74 out of 950 women without breast cancer but with a positive mammogram will have a false-positive sonogram. ^1^ Footnote: Assume for your calculations that the results of both tests are (statistically) independent for women with breast cancer as well as for women without breast cancer.	The probability of an HIV infection for a low-risk client is 0.01%. The probability that an HIV-infected client will have a positive ELISA test result is 99.9%. The probability that an HIV-infected client will have a positive Western Blot test result is 99.8%. The probability that a client without HIV infection will have a false-positive ELISA test result is 0.4%. The probability that a client without HIV infection will have a false-positive Western Blot test result is 0.1%. ^1^ Footnote: Assume for your calculations that the results of both tests are (statistically) independent for HIV-infected clients as well as for clients who are not HIV-infected.	100 out of 1,000,000 low-risk clients are HIV-infected. 100 out of 100 HIV-infected clients will have a positive ELISA test result. 100 out of 100 HIV-infected clients with a positive ELISA test result will have a positive Western Blot test result. 4,000 out of 999,900 clients without an HIV infection will have a false-positive ELISA test result. 4 out of 4,000 clients without an HIV infection but with a positive ELISA test result will have a false-positive Western Blot test result. ^1^ Footnote: Assume for your calculations that the results of both tests are (statistically) independent for HIV-infected clients as well as for clients who are not HIV-infected.
**Tree diagram**	Probability tree (in the tree-only and in the text-and-tree version)	Natural frequency tree (in the tree-only and in the text-and-tree version)	Probability tree (in the tree-only and in the text-and-tree version)	Natural frequency tree (in the tree-only and in the text-and-tree version)
**Question**	What is the probability that a woman with both positive mammogram and positive sonogram actually has breast cancer?	How many of the women with both positive mammogram and positive sonogram actually have breast cancer?	What is the probability that a client with both positive ELISA test and positive Western Blot test results is actually HIV-infected?	How many of the clients with both positive ELISA test and positive Western Blot test results are actually HIV-infected?
	Answer: _______	Answer: ____ out of ____	Answer: _______	Answer: ____ out of ____

#### Procedure

Each participant received one of the two problem contexts in probability format and the other problem context in natural frequency format, with the order of context and information format varied systematically. When one of the problems the participant worked on had a certain presentation of information (e.g., text only), the other problem contained one of the other remaining types of information presentation.

#### Solutions of the problems

The solution for the breast cancer screening problem is 76 out of 150, or about 50.7%. Note that the positive predictive value of about 50% corresponds to the actual values for women who participate in breast cancer screenings and receive positive results from a mammography as well as another non-invasive clarification (according to the latest evaluation report of the German Cooperative Association for Mammography [60]). For the HIV testing problem, the solution is 100 out of 104, or about 96.2%. Following Prinz et al. [6], the HIV testing problem uses a combined sensitivity (99.7%) and a combined specificity (99.9996%) of the ELISA test and the Western Blot test, resulting in a positive predictive value of about 96% when a prevalence of 0.01% is assumed (see also [61]).

It should be noted that in the medical 2-test case, the problem of conditional independence arises (see Footnote 1 in the Text section of Table 2). Readers interested in details concerning this issue can find more information in the S1 Appendix.

#### Coding

In accordance with Gigerenzer and Hoffrage [18], we classified a response elicited from a probability version as correct if it was the exact Bayesian solution or rounded to the next whole percentage point above or below (i.e., in the breast cancer screening problem, all solutions between 50% and 51%, and in the HIV testing problem, all solutions between 96% and 97% were classified as correct). In the natural frequency versions, responses were classified as correct only if both numbers (e.g., in the breast cancer screening solution of “76 out of 150”, both the 76 and the 150) were denoted correctly (a very conservative criterion regarding the natural frequency version; see also [28]).

#### Administration

Students were examined in larger groups during their university lecture sessions. Trained administrators guaranteed a quiet atmosphere and professional supervision of the study. Students sitting next to each other always worked on different versions. Pocket calculators were distributed and students were allowed to use them at any point during the test. There were no time constraints for completing the questionnaire. Participants needed on average about 30 minutes total for both tasks.

### Results

**Fig 3:**
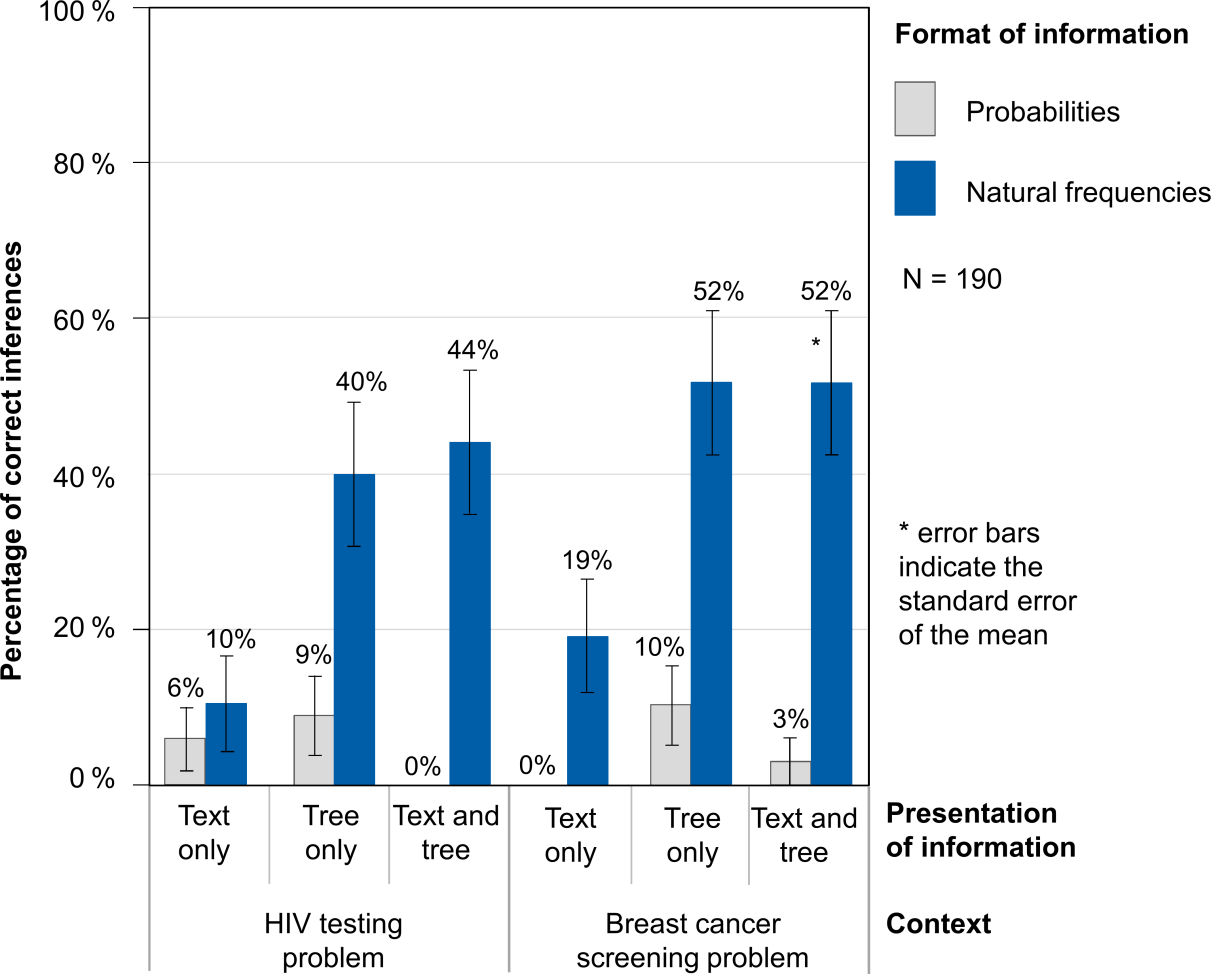
Percentages of correct inferences in Study 1.

Study 1 yielded two important findings (Fig 3). First, students performed better when statistical information was presented in natural frequencies (36% correct inferences across context and presentation) rather than as probability versions (5% correct inferences across context and presentation). This finding holds true for both contexts and for all three presentation formats. Second, the addition of a tree diagram leads to higher performance rates (again holding true across all versions and conditions). One exception is the weaker performance observed with the probability format in the HIV testing problem, which went from 6% with the text-only version to 0% with the text-and-tree version. However, we refrained from statistically comparing performance rates in probability versions because of the low achievement in all of these versions. Interestingly, in the natural frequency format, performance did not differ between tree-only and text-and-tree versions (i.e., when a frequency tree is provided, the additional text is neither harmful nor helpful).

Note that the advantage of “tree versions” (i.e., tree-only versions or text-and-tree versions) over text-only versions is much stronger with respect to natural frequency trees (47% vs. 15% correct inferences across both contexts) than it is with respect to probability trees (6% vs. 3%; see Fig 3). However, the weaker results obtained with probability trees could be instructive, since probabilities and probability trees are frequently used in statistical textbooks in both secondary schools and universities. Furthermore, participants performed descriptively slightly better in almost every version of the breast cancer screening problem (23% correct inferences) than in the respective versions of the HIV testing problem (18% correct inferences).

Since probability trees obviously do not foster insight within Bayesian reasoning situations, we will concentrate in the following on the results of the natural frequency versions. In order to analyze the effect of tree diagrams in natural frequency versions, we ran a generalized linear mixed model with a logit link function. In this model we specified the text-and-tree version as the reference version and included the possible explanatory factors “omitting tree” (i.e., text-only version) and “omitting text” (i.e., tree-only version) to predict the probability of a correct inference.

According to the results of the generalized linear mixed model, the probability of solving the text-and-tree version was 47.7% (unstandardized regression coefficient: b_0_ = -0.09). The (unstandardized) regression coefficient for omitting the tree was significant (b_1_ = -1.68, SE = 0.44, z = -3.84, p < 0.001), suggesting that the probability for solving the text-only version is reduced to only 14.5%. In contrast, omitting the text (i.e., using the tree-only version) leads to a non-significant regression coefficient (b_2_ = -0.07, SE = 0.35, z = -0.19, p = 0.85), which implies that the probability of solving tree-only versions (46.0%) does not differ significantly from the probability of solving text-and-tree versions.

A closer inspection of the data revealed an additional effect of student high school’s grade point average (the German *Abiturnote*). However, implementing grade point average in the generalized linear mixed model did not change the presented results substantially (omitting the tree diagram was still a significant factor and omitting the text was still non-significant). In order to exclude possible transfer effects (learning from the first task for the second task), we also implemented the position number of the task as an additional factor in the generalized linear mixed model. However, it turned out that participants performed even slightly (but not significantly) better if a particular task was located at the first position, which allowed us to exclude a possible transfer effect. Notably, when a tree diagram was provided, several participants marked the branches relevant to the question, which leads directly to Study 2.

## Study 2

**Fig 4:**
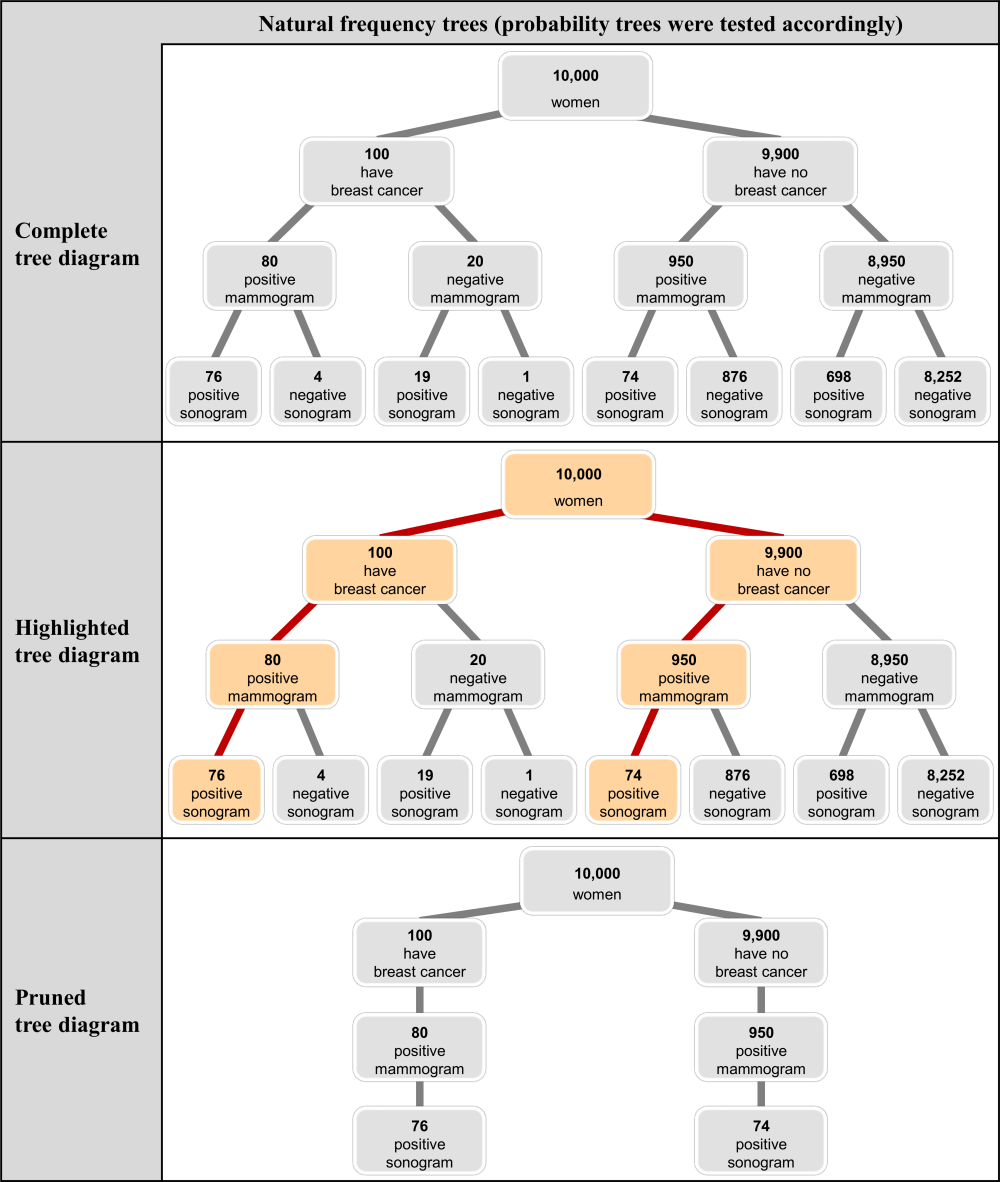
Three different tree diagrams with natural frequencies for the breast cancer screening problem (implemented in Study 2).

In the second study, we aimed to increase participant performance even more by providing different kinds of tree diagrams, that is, by highlighting the question-related branches in a special color or by pruning all branches but question-related ones. The three different tree diagrams that were implemented with respect to the breast cancer screening problem are shown in Fig 4. The respective probability versions of these tree diagrams were also tested in Study 2, of course.

### Method

#### Participants

In all, 198 medical students (65 men, 133 women) at different stages of their medical education were recruited in 2016 from University Hospital Regensburg. Students who participated in Study 1 were excluded from taking part in Study 2. Participants’ ages ranged from 18 to 38 years (*M* = 21.7, *SD* = 3.3). Again, all students were informed that their participation was voluntary and that anonymity was guaranteed. Participants had given their prior written consent to participating in the study. The Review Board of University Hospital Regensburg confirmed that no ethical approval would be necessary.

#### Design and materials

A paper-and-pencil-questionnaire contained two successive Bayesian tasks (both 2-test cases). We used the same medical contexts (breast cancer screening and HIV testing) as in Study 1 in order to enable comparisons between Study 1 and Study 2. We implemented a 3×2×2 design with the factors *kind of tree diagram* (complete tree vs. highlighted tree vs. pruned tree), *information format* (probabilities vs. natural frequencies), and *context* (breast cancer screening problem vs. HIV testing problem) (see Table 3 and section “Procedure and administration”).

**Table 3:** Design of the twelve resulting problem versions implemented (Study 2).

		Context	
		Breast cancer screening problem	HIV testing problem
**Information format**	Probabilities	**Kind of tree diagram** • Complete tree • Highlighted tree • Pruned tree	**Kind of tree diagram** • Complete tree • Highlighted tree • Pruned tree
	Natural frequencies	**Kind of tree diagram** • Complete tree • Highlighted tree • Pruned tree	**Kind of tree diagram** • Complete tree • Highlighted tree • Pruned tree

Note: In Study 2, the textual information was provided in each version.

In light of the results obtained in Study 1, it had to be decided whether or not the statistical information should be presented in text form as well. Because the text-and-tree version produced the strongest student performance in Study 1, we decided to use this version in Study 2 as well in order to be conservative when investigating the beneficial effects of highlighting and pruning tree diagrams.

All versions began with the same medical situations used in Study 1. After the statistical information was provided, one of the three different kinds of tree diagrams was presented. Finally, the question was provided in the same format as the information in the text. Note that the complete-tree versions in Study 2 were identical to the text-and-tree version in Study 1.

#### Procedure and administration

As in Study 1, each participant received one of the two problem contexts in probability format and the other in natural frequency format, again with the order of problem context and information format varying systematically. This time, the two problems each participant worked on had two out of the three different kinds of tree diagrams: *complete tree*, *highlighted tree* and *pruned tree*. For further details of the study administration, see Study 1.

#### Solutions of the problems and coding

Since Study 1 and Study 2 did not differ in these two aspects, the respective solution and coding can be taken from Study 1. Again, readers interested in the issue of conditional independence can consult S1 Appendix.

### Results

**Fig 5:**
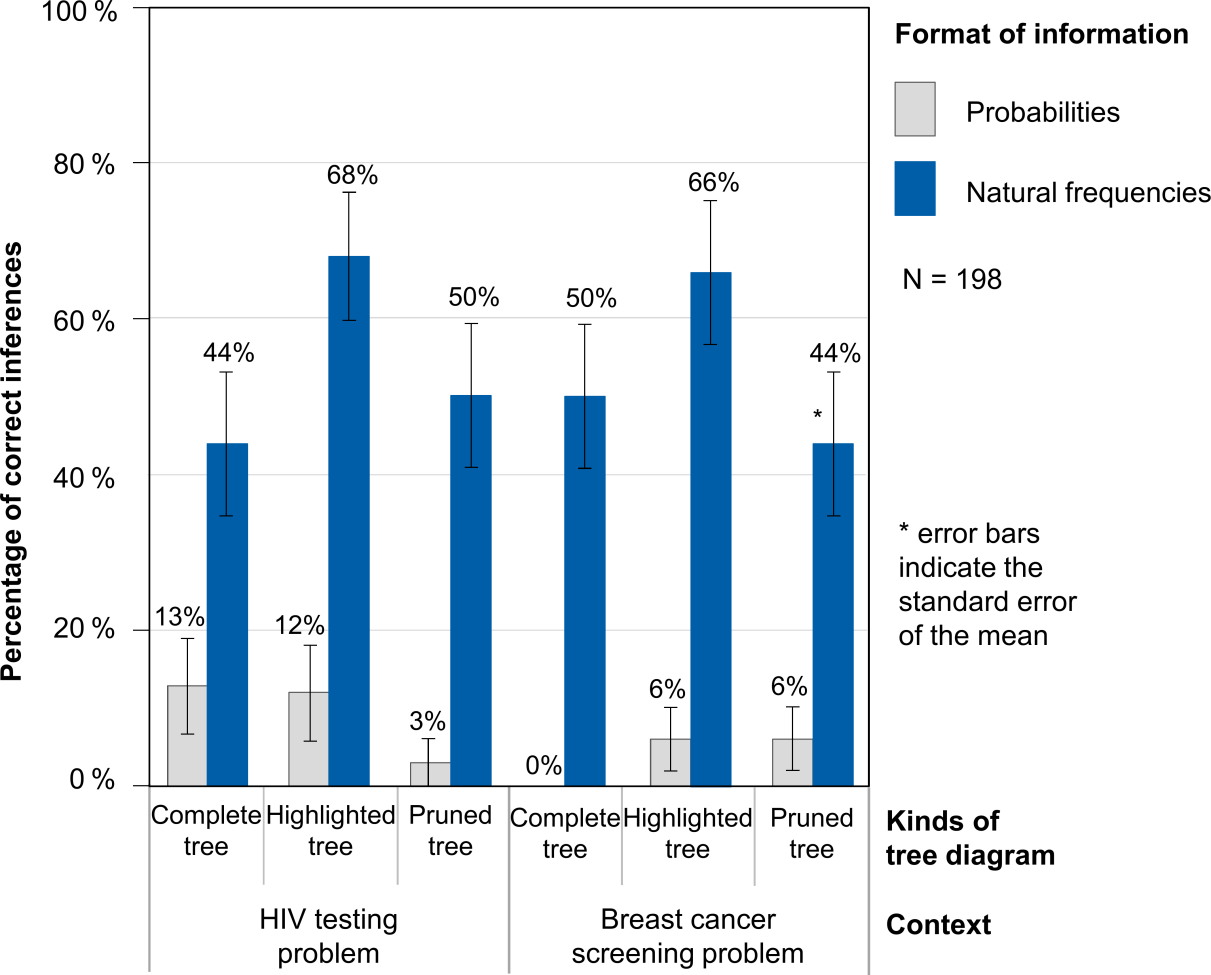
Percentages of correct inferences in Study 2.

Study 2 produced three important findings (Fig 5). First, as in Study 1, student performance was substantially stronger when the statistical information in the problem was presented in natural frequencies (54% correct inferences across contexts and kinds of tree diagram) rather than probabilities (7% correct inferences). Because probability trees in Study 2 also did not constitute helpful visualizations (the maximum was 13% correct solutions; see Fig 5), we concentrate on natural frequency trees here again. Second, highlighting the two relevant branches of natural frequency trees leads to the highest performance rates, namely 67% (across contexts) as compared to 47% with the complete tree (not highlighted). Third, the use of a pruned tree does not improve Bayesian reasoning more than the use of a complete tree (both performance rates were 47% across contexts).

In order to analyze the effect of different kinds of tree diagrams in natural frequency versions, we again ran a generalized linear mixed model with a logit link function. In this model we specified the complete-tree version as the reference version (this version is identical to the text-and-tree version in Study 1) and included the possible explanatory factors *highlighting tree* and *pruning tree* to predict the probability of a correct inference.

According to the results of the generalized linear mixed model, the probability of solving the complete-tree version was 46.9% (unstandardized regression coefficient: b_0_ = -0.13). The (unstandardized) regression coefficient for the highlighted tree was significant (b_1_ = 0.82, SE = 0.36, z = 2.26, p = 0.02), suggesting that the probability for solving a version with a highlighted tree increased to even 66.7%. In contrast, pruning the irrelevant branches of the tree diagrams leads to a non-significant regression coefficient (b_2_ = 0.01, SE = 0.35, z = 0.02, p = 0.98), which implies a probability for solving the task of 47.1% (comparable to the complete-tree version). In summary, highlighting the relevant branches and simultaneously presenting the complete situation can foster insight.

Moreover, in Study 2 there was an additional effect of the position number of the solved tasks. All versions placed in the first position were again solved better than the identical tasks placed in the second position. In contrast to Study 1, this factor was even significant. However, implementing the position number of the task in the generalized linear mixed model did not change the presented results substantially (highlighting the tree diagram was still a significant factor and pruning the tree was still non-significant). Therefore, we can again exclude transfer effects in Study 2. Whereas in Study 1 grade point average but not position number had a significant effect, the opposite was the case in Study 2 (however, both effects did not affect the main results). Furthermore, *context* (which was not a factor of interest) did not change the results substantially in either study.

Because the text-and-tree versions of Study 1 were identical to the complete-tree versions of Study 2 (each for both contexts and both formats as well), and since the performance of the participants in these versions was comparable in both studies, we assume similar competencies in both subsamples. Therefore, it seems reasonable to compare performances between the two single studies.

## Recommendations for fostering insight

Taken together, Studies 1 and 2 suggest three strategies that can be recommended to stimulate insight within Bayesian reasoning situations: (1) replace probabilities by natural frequencies, (2) create a natural frequency tree, and (3) highlight the two question-relevant branches in the natural frequency tree.

## General discussion

Both studies (1) replicated earlier findings that—regardless of visualization—natural frequency versions could be solved much more easily than probability versions (e.g., for 1-test cases see [18,19,47,62], and for 2-test cases see [51]). The new results demonstrated that (2) natural frequency trees but not probability trees were substantially helpful and that (3) highlighting the question-related branches in a natural frequency tree can additionally improve performance, but pruning the tree does not.

Since in all implemented probability versions participant performance ranged from 0% to only 13% (across both studies), probability tree diagrams clearly do not qualify as visualizations that stimulate great insight within Bayesian reasoning situations. Because the focus of the present article is not the reinvestigation of format effects (the probability versions only served as control versions) but the boosting of participant performance, we will concentrate in the remaining discussion on natural frequency trees.

Considering the Cognitive Load Theory [53] and the Cognitive Theory of Multimedia Learning [54], two results here are remarkable: (1) text-and-tree versions and tree-only versions (Study 1) can both be solved with similarly little effort, and (2) pruning irrelevant branches (Study 2) does not help participants, probably because the situation as a whole is no longer fully presented. Neither finding supports the hypothesis that the extraneous cognitive load is increased by (a) presenting text and tree simultaneously or (b) presenting information that is not directly relevant to the question at hand. Yet highlighting the question-related branches (while still showing the irrelevant branches) was of greatest help for participants in Bayesian reasoning situations, therefore supporting the signaling principle with respect to frequency trees [58,59].

Thus, highlighted natural frequency trees are the best recommendation for teaching statistics (in secondary schools and at universities) and for communicating risks (e.g., in the medical domain). With respect to medical decision making, understanding the meaning of medical test results is crucial for medical students and physicians as well as for patients, because it can reduce the possible harms of overdiagnosis and overtreatment but can also reduce the danger of serious diseases being overlooked.

Frequency trees can easily be constructed and, if need be, also extended to situations with more than one hypothesis (e.g., several possible diseases), to non-binary test results or symptoms (e.g., unclear test results or symptoms), or to situations where even more than two tests (or symptoms) are provided [51]. Furthermore, besides the described *causal trees* (first split the sample into patients with the disease and without the disease and then split these two nodes into sets with respect to the test result), *diagnostic trees* including natural frequencies can be constructed (i.e. first split the sample with respect to the test result and then with respect to the disease) [63–65].

Interestingly, in both studies, performance did not depend on the students’ level of medical education, which indicates that statistical education is not sufficiently implemented in the training of medical students. However, it has to be noted that we did not run a training study, and thus our results suggest that natural frequency trees are effective even in the absence of prior instruction. Consequently, natural frequency trees can be directly used by patients and physicians and hence should be implemented in medical textbooks and in statistics education materials for prospective physicians, thus making this helpful communication tool available to both physicians and patients.

## Supporting information

S1 Appendix Conditional independence. (DOCX)
