# Supplementary material for: Visualizing the Bayesian 2-test case: The effect of tree diagrams on medical decision making
Source: PLoS One. 2018 Mar 27;13(3):e0195029. doi: 10.1371/journal.pone.0195029 (PMC5871005; doi:10.1371/journal.pone.0195029)
Supplement: S1 Appendix — (DOCX) [file pone.0195029.s001.docx]

**S1 appendix.** **Conditional independence.**

**Conditional independence**

**Alternative presentations of the 2-test case**

An alternative way to present statistical information in a 2-test case were providing the sensitivity or the specificity of the whole testing procedure (e.g., a combined sensitivity and a combined specificity), which would reduce it to a 1-test case. However, in the literature on evidence-based medicine, sensitivities or specificities are usually only provided for a single test, and there is little available information about combined sensitivities or combined specificities because, in most medical test procedures, there is no standard test sequence that is followed consistently (further possibilities to extent the 1-test case to the 2-test case can be found in [1]).

If sensitivities and specificities are presented for each single test in the 2-test case, the question of the conditional independence of the involved tests arises. Because in probability theory conditional independence is based on the simpler situation of a statistical independence of two events, we first address the latter concept.

**Statistical independence of two events**

Note that the two events *A* and *B* are (statistically) independent if and only if $P\left( A\cap B \right)=P\left( A \right)\cdot P(B)$ or, equivalently, $P\left( A | B \right)=P(A)$. This means that the occurrence of one event does not affect the probability of the occurrence of the other event.

Also note that in the 1-test case, disease and test results (both considered events) are obviously statistically dependent, which is why this is not an issue in the 1-test case.

**Conditional independence of two events given a third event**

For three events the question of conditional independence arises. The two events *A* and *B* are conditionally independent *given C* if and only if

$$P\left( A\cap B | C \right)=P\left( A | C \right)\cdot P(B|C)$$

This is equivalent to

$$P\left( A | B\cap C \right)=P\left( A | C \right)=P\left( A | \neg B\cap C \right)$$

and to

$$P\left( B | A\cap C \right)=P\left( B | C \right)=P\left( B | \neg A\cap C \right)$$

Thus conditional independence of the events *A* and *B* given *C* means that the probability of *A* given *C* is not affected by the simultaneous occurrence of the event *B* (or *not B*). The same holds true if *A* and *B* are interchanged.

Note that it is possible that the events *A* and *B* are conditionally independent given *C*, but not conditionally independent given the complementary event *not C*.

**Conditional independence in medical Bayesian 2-test cases**

In our study, the question of the conditional independence of the two test results occurs both in the case of healthy people and in the case of people with a certain disease *D*. The conditional independence of the two test results

1. given that the person has the disease (*D*) means

$$P\left( T2+ | T1+\cap D \right)=P(T2+|D)$$

1. given that the person does not have the disease means

$$P\left( T2+ | T1+\cap\neg D \right)=P(T2+|\neg D)$$

Consequently, depicting the original sensitivity of the second test directly at the lower branch of a probability tree is only possible in the case of the conditional independence of both test results involved for people with the disease.

**Medical reality**

In real-life medical situations, it cannot be assumed as a matter of course that two successive medical tests are conditionally independent both for individuals with the disease and individuals without the disease: For instance, a false-positive HIV test result might occur because of a chronic hepatitis B infection. Imagine that a person who is not HIV infected but who is infected with hepatitis B receives a (false!) positive test result from an HIV test because of the hepatitis B. If a second HIV test is then conducted, the probability that the second test will also be positive is higher than if there has been no first positive test result, because the reason for the false-positive result also exists in the second test.

As a consequence, when both tests applied are the same (e.g., a second ELISA test given after a positive ELISA test result), or are at least based on similar medical methods, conditional dependence should be assumed. However, the less similar the two test procedures are, the more likely they are not to affect each other (e.g., see [2]).

Concerning the contexts implemented in our study, the medical students we observed would assume the “independence” of both test procedures (probably without being able to provide a formula or distinguish between “independence” and “conditional independence”). Interestingly, the conditional independence of medical tests seems not to be considered an important issue in the field of medicine today.

**Experimental decisions for our study**

*How should the natural frequency version look?*

According to the natural sampling paradigm [3], the textual information in our frequency versions should match the sampling process that physicians utilize in real-life situations, namely performing one test after the other in a typical sequence. Thus the absolute numbers sampled represent the sensitivity and the false-alarm rate in an ecologically valid way. These obtained absolute numbers are perfectly represented by the sequential partitioning of patients in the textual natural frequency versions and in the tree diagram as well. Therefore, both our 2-test case textual formulation and the tree diagrams with natural frequencies grow out of the idea of the natural frequency approach of Gigerenzer and Hoffrage [4].

*How should the probability version look?*

We declined to present the sensitivity and the false alarm rate of the second test in the textual formulation as conditional upon the result of the first test for the following reasons.

In a pilot study, we tried to implement the information, for example, presenting the sensitivity of the sonography as conditional upon the mammography test result (“The probability that a woman with breast cancer will have a positive sonogram, given that she has already had a positive mammogram, is 95%” as compared to “The probability that a woman with breast cancer will have a positive sonogram is 95%”). However, this led to even weaker performance because participants seemed to assume conditional independence anyway. For them, the supplement was misleading because stressing this condition suggests that the sensitivity of the second test would differ for different results of the first test. And if an event occurs with the same probability when given “A” as when given “not A”, what is the point of mentioning “given A”?

In addition, there is another theoretical reason for not presenting combined sensitivities or sensitivities bases on previous test results: In evidence-based medicine, sensitivities, false alarm-rates, and specificities are almost never provided as conditional on other tests. As mentioned above, it is virtually impossible to find statistical information on sensitivities or specificities presented as conditional on previous test results. Therefore, our wording choice strengthens the ecological validity for the examined medical students.

However, in order to be mathematically correct, we added footnote 1 in all versions implemented in both studies (see Table 3).

References

1. Binder K, Krauss S. Generalizations of the Bayesian reasoning paradigm. submitted.

2. Shen Y, Wu D, Zelen M. Testing the Independence of Two Diagnostic Tests. Biometrics. 2001; 57: 1009–1017. doi: 10.1111/j.0006-341X.2001.01009.x.

3. Kleiter GD. Natural sampling: Rationality without base rates. In: Fischer GH, Laming, D. R. J, editors. Contributions to Mathematical Psychology, Psychometrics, and Methodology. New York: Springer; 1994. pp. 375–388.

4. Gigerenzer G, Hoffrage U. How to improve Bayesian reasoning without instruction: frequency formats. Psychol. Rev. 1995; 102: 684–704. doi: 10.1037/0033295X.102.4.684.
